# Thermal behavior in compact-type cryogenic needle valve with integrated heat sinks

**DOI:** 10.1038/s41598-025-31069-z

**Published:** 2025-12-09

**Authors:** Su Jung Kang, Ju Hwan Kwak, Seung Ho Han

**Affiliations:** 1 Department of Mechanical Engineering, Dong-A University, Busan, Korea; 2 SGT Inc., Busan, Korea

**Keywords:** Engineering, Materials science

## Abstract

A cryogenic needle valve is used to precisely control the flow rate of working fluids such as liquid hydrogen and liquefied natural gas, making it essential to design the valve with consideration of the cryogenic environment. To prevent freezing of the packing materials, which directly affects leakage, extended bonnet-type valves are commonly used. In these designs, the bonnet length is increased to position the packing farther from the flow path. However, due to spatial constraints, extended bonnet structures are often unsuitable for installation in confined spaces. To address this limitation, a compact-type valve was developed by significantly shortening the bonnet length and incorporating a heat sink capable of providing the required heat transfer performance within the reduced length. In this study, a cryogenic environment test using liquid nitrogen, along with heat transfer analysis, was conducted on the compact-type cryogenic needle valve to evaluate the thermal behavior of its components. A parametric study and size optimization were performed by treating the heat sink’s outer diameter and thickness as shape design variables, with the goal of minimizing its size while maintaining the necessary heat transfer performance in the shortened bonnet. As a result of the optimization, the heat sink volume was reduced by approximately 54% compared to the initial design. A cryogenic leakage test was subsequently conducted on a prototype equipped with the optimized heat sink, in accordance with BS6364, confirming that no leakage occurred.

## Introduction

A cryogenic needle valve is used for precise fluid flow control in applications that require accurate regulation, particularly when handling cryogenic fluids. Cryogenic fluids such as liquid hydrogen (LH₂) and liquefied natural gas (LNG) are typically operated at temperatures ranging from − 253 °C to − 162 °C^1–3^. At these extremely low temperatures, hardening of the packing materials can alter gasket tolerances, leading to potential leakage^4,5^. To mitigate this, an extended bonnet structure is commonly employed to position the packing away from the flow path, thereby reducing the risk of packing hardening^6,7^. However, the increased length of the extended bonnet can pose significant challenges in confined installation spaces. Therefore, it is essential to explore design strategies that minimize bonnet length while effectively preventing packing freeze.

Studies investigating the reduction of extended bonnet length through consideration of thermal behavior have been conducted. Jeong et al.^8^ performed thermal and stress analyses on a globe valve for LNG applications to evaluate the effects of temperature and pressure on bonnet length and thickness. They reported that increasing the bonnet length and decreasing its thickness caused the temperature at the packing region to approach ambient temperature, while stress due to internal pressure and thermal deformation increased. Jeon et al.^9^ conducted a thermal analysis to optimize the bonnet length of a cryogenic gate valve. Their results, obtained for four bonnet lengths ranging from 80 mm to 480 mm at − 162 °C, showed that longer bonnets led to higher packing temperatures, reaching 11.5 °C at a length of 480 mm. The optimal bonnet length to prevent packing freeze was determined to be 160 mm. These studies primarily focused on evaluating the thermal characteristics of extended bonnet valves and assessing their structural integrity under thermal deformation induced by cryogenic fluids. By parameterizing the bonnet’s length and thickness, suitable dimensions were derived to prevent packing freeze and leakage. Nevertheless, even after thermal optimization, the requirement for a relatively long extended bonnet poses challenges in confined installation environments. In particular, further reduction of the bonnet length was essential for practical implementation in space-limited system, revealing the inherent of the conventional extended bonnet design.

To address this issue, further investigations were needed to explore alternative design approaches that can reduce bonnet length while maintaining or improving thermal performance. An alternative design strategy includes the incorporation of a drip-pan structure to increase the heat dissipation area and prevent packing damage caused by low temperatures^10^. Li et al.^11^. reported that in various types of cryogenic valves, a drip plate structure has been applied to increase heat dissipation and prevent packing damage caused by low temperatures. They proposed a method to reduce the thermal impact of cryogenic fluids transmitted to the stem through conduction and convection by attaching a drip plate to the bonnet of cryogenic valves. A case study was conducted in which the height, radius, and thickness of the drip plate were considered as shape design variables, and the packing temperature—critical for preventing leakage—was evaluated.

However, since the drip plate mainly acts as a passive structure that limits condensate flow rather than actively regulating heat transfer, its effectiveness in maintaining the packing temperature is limited. To overcome this limitation, the present study introduces a pair of heat sinks—serving as a thermally enhanced structure extended the concept of the conventional a drip plate—were installed on the upper and lower parts of the stem, with the goal of reducing the bonnet length. Numerical analysis and experiments were performed to evaluate the thermal behavior of the compact-type cryogenic needle valve. Additionally, a size optimal design was performed by parameterizing the outer diameter and thickness of the heat sinks, and prediction models for the packing temperature were induced using Kriging and artificial neural network. This approach enabled both the prevention of actuator freezing and leakage occurring at packing gland, and the size optimization of the valve design for weight reduction.

## Compact-type needle valve with integrated heat sinks

**Fig. 1:**
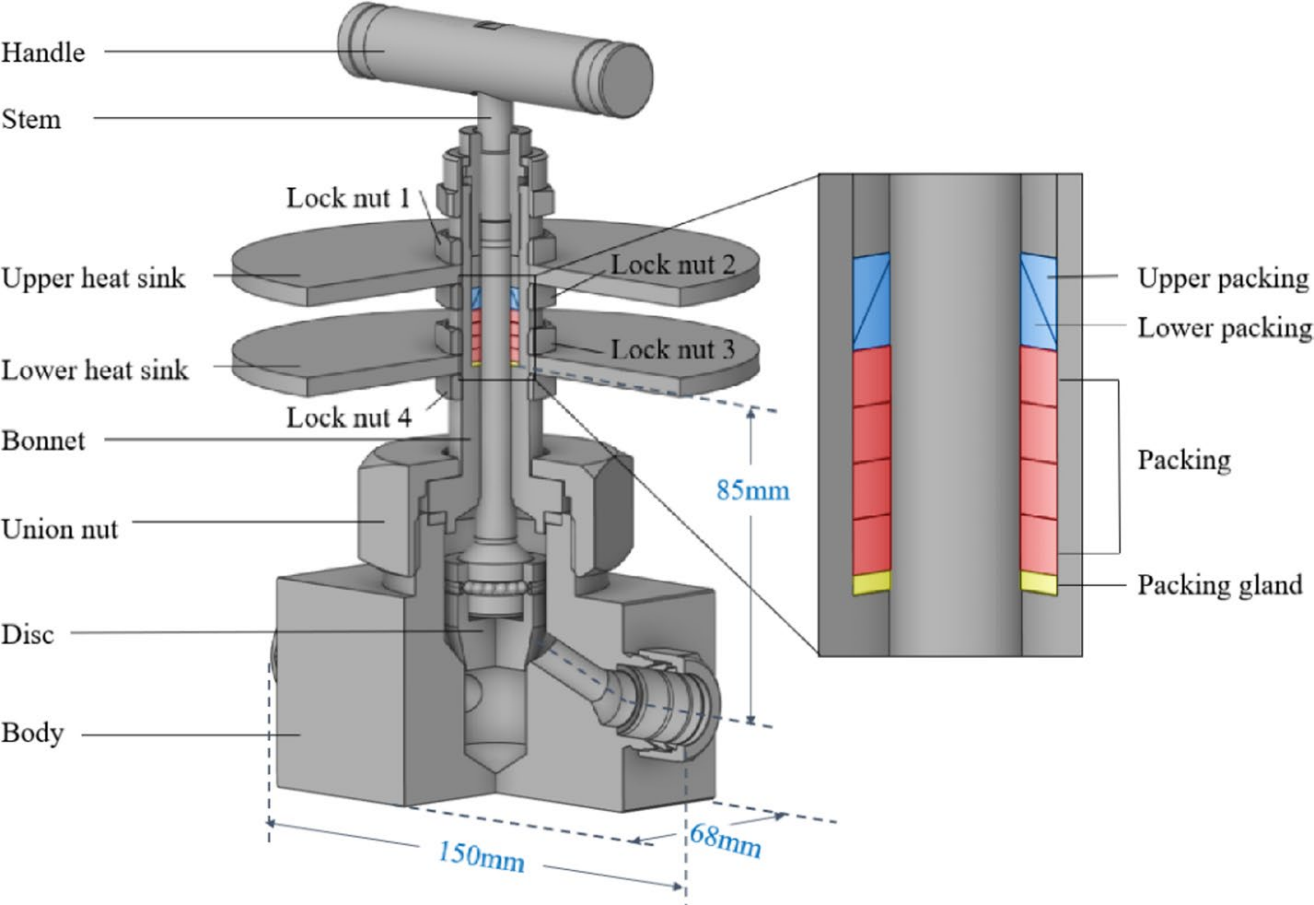
Cross-section view of compact-type needle valve with integrated heat sinks.

The compact-type needle valve, as shown in Fig. 1, is composed of a body, bonnet, union nut, disc, stem, handle, packing, and a pair of heat sinks. According to BS 6364^12^, for a 3/4-inch cryogenic valve, the required minimum distance from the flow path to the packing is 500 mm. However, in the compact-type needle valve, this distance was reduced to 85 mm, significantly shortening the bonnet length compared to conventional cryogenic valves. The heat sinks enhanced convective heat transfer by increasing the contact area with ambient air, thereby preventing the handle and packing from freezing. To ensure that the overall length of the valve did not exceed the face-to-face dimension of 150 mm, the outer diameter of each heat sink was determined to be 150 mm. The thickness was defined as 5 mm, corresponding to the minimum machinable dimension for manufacturability, and a volume of approximately 86,052 mm^3^ was obtained per disk. When only a single heat sink was attached, the packing temperature was found to exceed the limit operating temperature of − 70 °C, indicating a potential risk of freezing. Consequently, the pair of disk-shaped heat sinks was installed on the outer surface of the bonnet to improve heat transfer performance. The heat sinks were positioned at the level of the packing section so that ambient heat could be effectively delivered to the sealing region and the packing temperature could be maintained at a stable level.

The materials used in the compact-type needle valve and their respective properties are listed in Table 1. All components, except for the packing, were made from SUS 316 used commonly in cryogenic environments. The heat sinks were also fabricated from SUS 316 to match the coefficient of thermal expansion, thereby minimizing localized thermal stresses at the joints.

Meanwhile, the packing material, applied to prevent leakage, was made of high-thermal-conductivity graphite to reduce the risk of failure due to localized thermal stress concentration. The upper and lower packing elements, along with the packing gland, were made of low-thermal-conductivity materials—Polychlorotrifluoroethylene (PCTFE) and Polyetheretherketone (PEEK)—to suppress the transfer of cryogenic heat to the handle.

**Table 1:** Materials and their properties for compact-type needle valve with integrated heat sinks.

Parts	Materials	Thermal expansion coefficient [/K]	Thermal conductivity [W/(m·K)]
Body	SUS 316	1.6 × 10^–5^	14.3
Bonnet			
Disc			
Handle			
Heat sink			
Stem			
Union nut			
Packing	Graphite	2.9 × 10^–6^	138.6
Packing gland	PEEK	4.4 × 10^–5^	0.25
Upper/Lower packing	PCTFE	7.0 × 10^–5^	0.20

## Thermal behavior in compact-type needle valve

### Cryogenic environment test

To estimate the heat transfer characteristics of the compact-type needle valve, a cryogenic environment test was conducted. Liquid nitrogen was used as the working fluid, and temperatures at various points on the valve were measured to observe thermal behavior resulting from heat transfer caused by the liquid nitrogen.

**Fig. 2:**
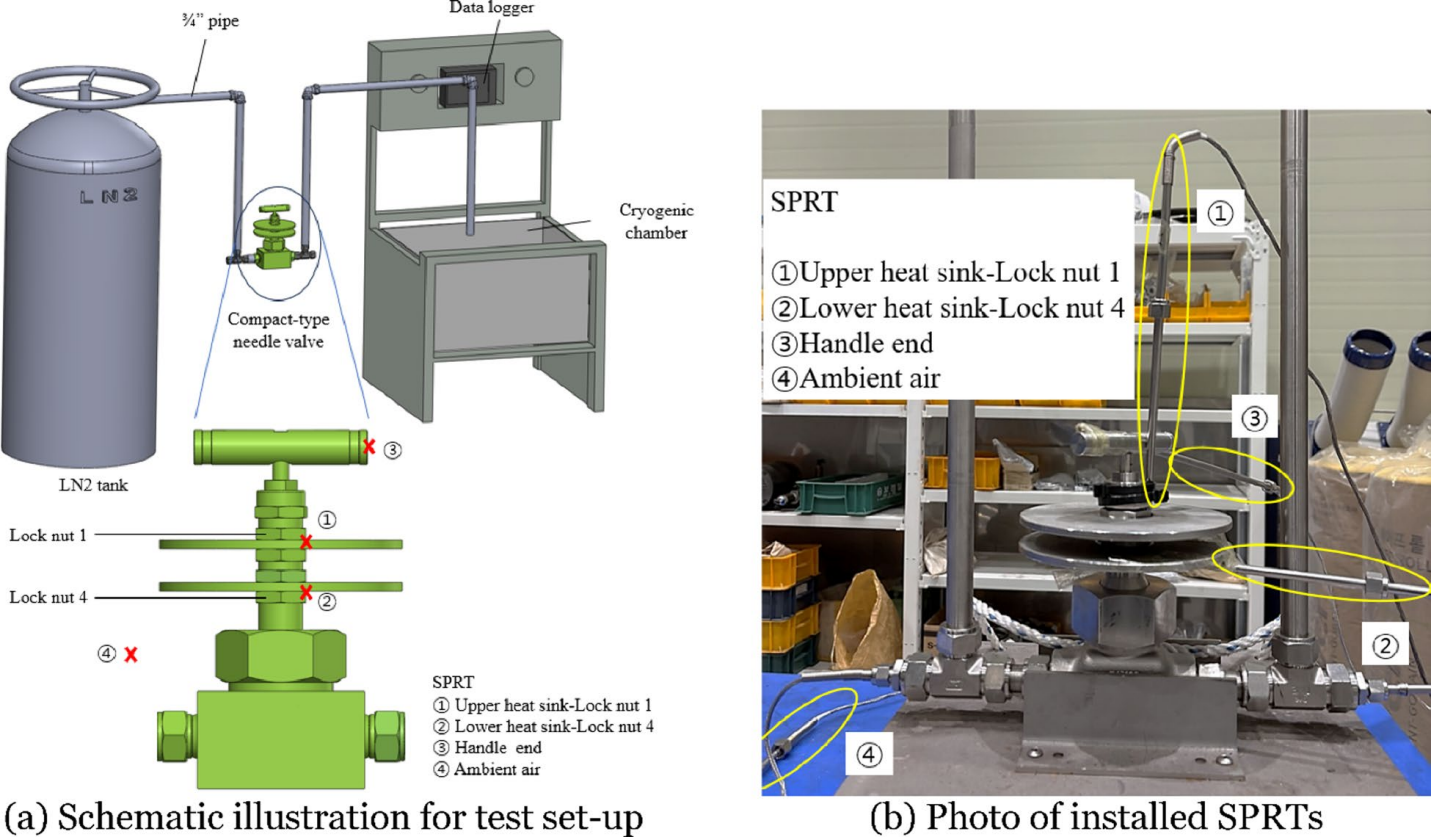
Test set-up for cryogenic environment test.

Figure 2 shows the test set-up and temperature measurement equipment used for the cryogenic environment test. Figure 2a is a schematic illustration of the test set-up, where the compact-type needle valve was installed between a liquid nitrogen storage vessel and a cryogenic chamber, connected via 3/4-inch diameter piping. Temperatures were measured at three locations on the valve and in the ambient air. Figure 2b shows the temperature measurement locations and the temperature sensors used. The temperature sensors were standard platinum resistance thermometers (SPRT), specifically the AM1860-25 model by ACCUMAC, which operated based on resistance values generated when current was applied. They were suitable for use under cryogenic environment bellow down to − 200 °C. A data logger from GRAPHTEC was used for real-time temperature measurement and recording, and a circuit was constructed using a thermometry bridge to connect to the SPRTs. Prior to testing, the measurement system was calibrated using a temperature calibrator from Fluke to ensure accuracy and traceability. Table 2 presents the specifications of the equipment used for temperature measurement. The SPRTs were installed at four specific locations on the compact-type needle valve, as follows: ① the contact point between the upper heat sink and lock nut 1, ② the contact point between the lower heat sink and lock nut 4, ③ the handle end, and ④ the ambient air.

**Fig. 3:**
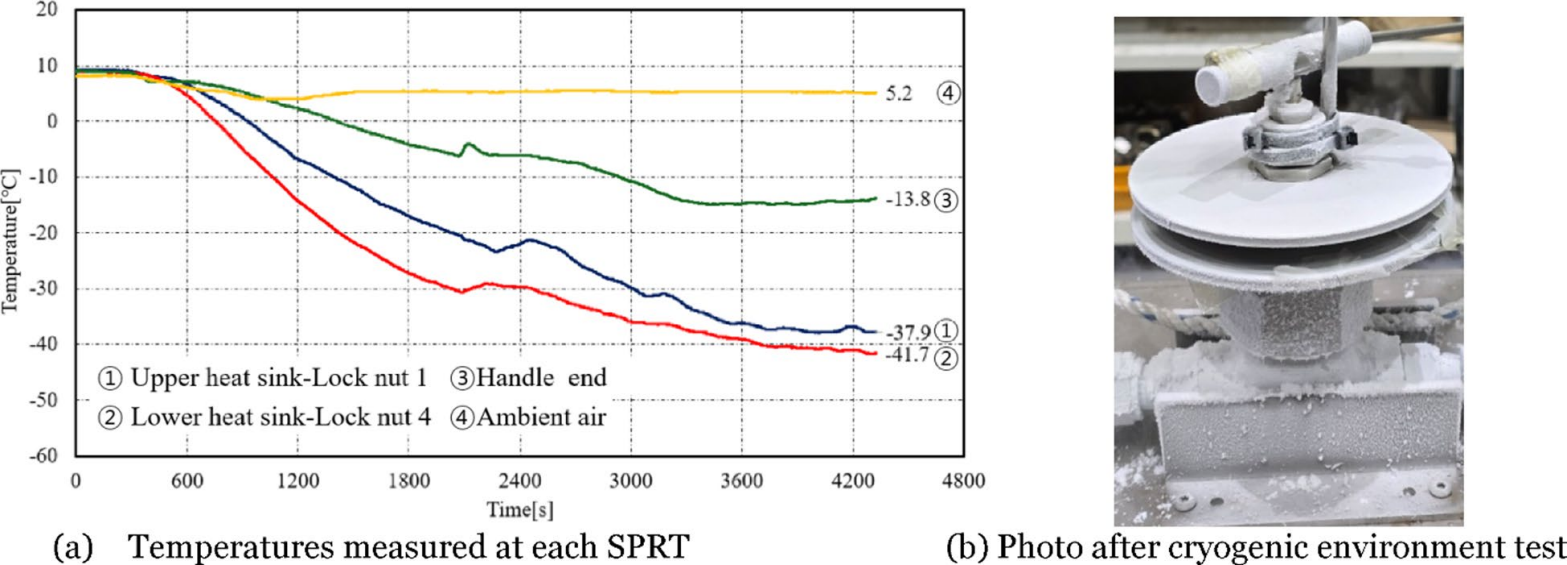
Experimental results from cryogenic environment test.

The cryogenic environment test was conducted over a total duration of 1 h and 10 min, with the valve fully open and liquid nitrogen introduced at the inlet. Temperatures at each measurement point were recorded at 1-second intervals. Figure 3a shows the results of temperature measurement at the four measurement points. The test began at an ambient lab temperature of approximately 10 °C, and the ambient air temperature remained stable at 5.2 °C, as seen in ④, throughout the experiment. At the handle end (③), the temperature stabilized at − 13.8 °C after 1 h. The temperatures at the contact points between the upper and lower heat sinks and the lock nuts (① and ②) were significantly affected by the liquid nitrogen, continuously decreasing throughout the test and reaching bellow down to − 37.9 °C and − 41.7 °C, respectively. After the cryogenic environment test was completed, the compact-type cryogenic needle valve appeared to be entirely frozen, as shown in Fig. 3b.

**Table 2:** Equipment of temperature measurement.

Equipment	Specifications	
SPRT	Model	ACCUMAC, AM1860-25
	Measurement range	–200 °C to 670 °C
	Nominal resistance at 0 °C	25.5 Ω
	Accuracy	0.06 °C
Precision thermometry bridge	Model	ISOTECH, Micro K-800
	Measurement range	–200 °C to 1800 °C
	Accuracy whole range	0.8 ppm
Temperature calibrator	Model	Fluke, 754
	Measurement range	–200 °C to 100 °C
	Accuracy	0.07 °C
Data logger	Model	GRAPHTEC, GL840
	Input voltage range	20 mV to 100 V
	Accuracy	0.2 °C

### Heat transfer analysis

The heat transfer analysis was performed using the general three-dimensional heat conduction equation and the convection heat transfer relation. The governing equation for the finite element formulation was derived using the weighted residual method^13^.

The general transient heat conduction equation is expressed as:

$$\:\rho\:c\frac{\partial\:T}{\partial\:t}-\left(\frac{\partial\:}{\partial\:x}\left({k}_{x}\frac{\partial\:T}{\partial\:x}\right)+\frac{\partial\:}{\partial\:y}\left({k}_{y}\frac{\partial\:T}{\partial\:y}\right)+\frac{\partial\:}{\partial\:z}\left({k}_{z}\frac{\partial\:T}{\partial\:z}\right)\right)-\dot{{q}_{c}}=0$$

where, $\:\rho\:$ is the density of the used material, $\:c$ is the specific heat, $\:k$ is the coefficient of thermal conductivity, $\:\dot{q}$ is the internal heat generation rate per unit volume and unit time, and $\:T$ is the variable temperature for the coordinates, $\:x,\:y,\:z$ and $\:t$ denotes time. For stationary heat transfer, the previous equation becomes:

$$\:\left(\frac{\partial\:}{\partial\:x}\left({k}_{x}\frac{\partial\:T}{\partial\:x}\right)+\frac{\partial\:}{\partial\:y}\left({k}_{y}\frac{\partial\:T}{\partial\:y}\right)+\frac{\partial\:}{\partial\:z}\left({k}_{z}\frac{\partial\:T}{\partial\:z}\right)\right)=\dot{q}$$

Convection heat transfer relation is presented as:

$$\:{q}_{h}=h\left({T}_{s}-{T}_{\infty\:}\right)$$

where $\:{q}_{h}$ is the heat flux at the surface, $\:{T}_{s}$ is the surface temperature, $\:{T}_{\infty\:}$ is the ambient temperature, and $\:h$ is the convection heat transfer coefficient.

Therefore, governing equation for the finite element analysis can be formulated as follows:

$$\:\left[\left[{K}_{c}\right]+\left[{K}_{h}\right]\right]\left\{T\right\}=\left\{{Q}_{c}\right\}+\left\{{Q}_{Q}\right\}+\left\{{Q}_{q}\right\}+\left\{{Q}_{h}\right\}$$

where $\:\left[{K}_{c}\right]$ is the conduction matrix, $\:[{K}_{h}$] is the convection matrix, $\:\left\{T\right\}$ is the vector containing the nodal temperatures, $\:\left\{{Q}_{c}\right\}$ is the vector of heat flux due to conduction, $\:\left\{{Q}_{Q}\right\}$ is the vector of the heat generated, $\:\left\{{Q}_{q}\right\}$ is the vector of the specific heat, $\:{\{Q}_{h}\}$ is the vector of convection. Finite element equations for heat transfer problems can be obtained by applying the weighted residual method to the differential equation that governs the heat transfer problem.

The numerical analysis model based on the governing equation for the finite element analysis was constructed to evaluate the heat transfer characteristics of a compact-type cryogenic needle valve, and heat transfer analysis was conducted. The obtained results were compared with the results of the cryogenic environment test to validate the analysis methodology.

**Fig. 4:**
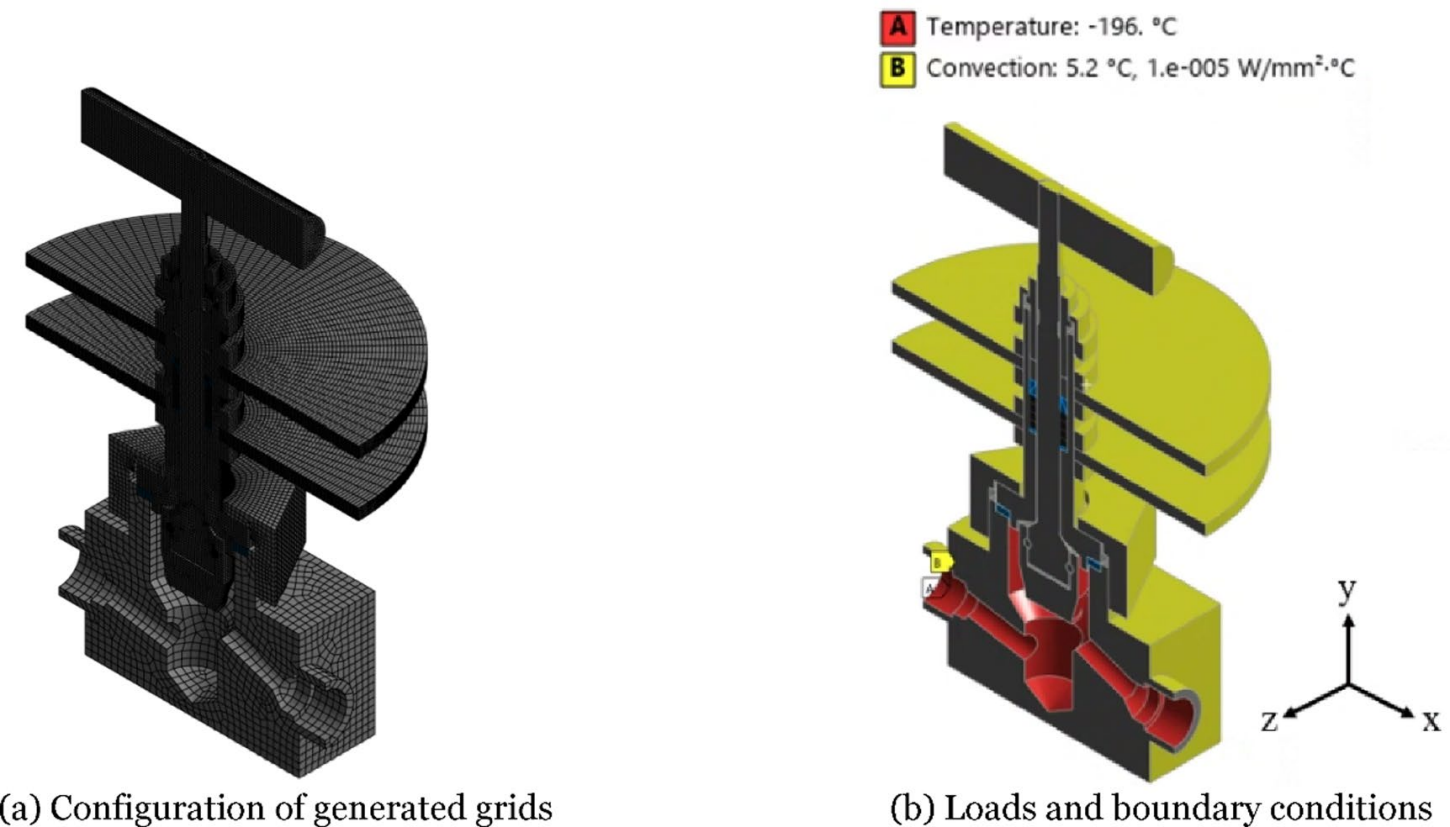
Pre-processing of heat transfer analysis.

The heat transfer analysis was conducted using the commercial software of ANSYS Workbench 2024 R2 Steady-State Thermal^14^. The temperature distribution of the compact-type needle valve under saturated conditions was estimated, where the temperature stabilized during the cryogenic environment test. The grid configuration, loads and boundary conditions for pre-processing of the heat transfer analysis are shown in Fig. 4. Figure 4a presents the overall grid configuration for the finite element model. Grids with hexahedron elements averaging 3 mm in size were generated, while a finer element of 1 mm was applied around the connection of heat sink and bonnet, packing, disc, and stem. The total number of elements and nodes in the finite element model are 182,964 and 810,841, respectively. Figure 4b shows the applied loads and boundary conditions used in the heat transfer analysis, which adopted a 1/2 symmetry condition. As in the cryogenic environment test, a thermal load condition of − 196 °C, corresponding to the temperature of liquid nitrogen, was applied to the flow path. The areas exposed to the atmosphere were set to the measured ambient temperature of 5.2 °C, and a heat transfer coefficient of 0.00001 W/mm²·°C under natural convection conditions^8^ was applied. The material properties of the valve components applied in the heat transfer analysis are presented in Table 1.

**Fig. 5:**
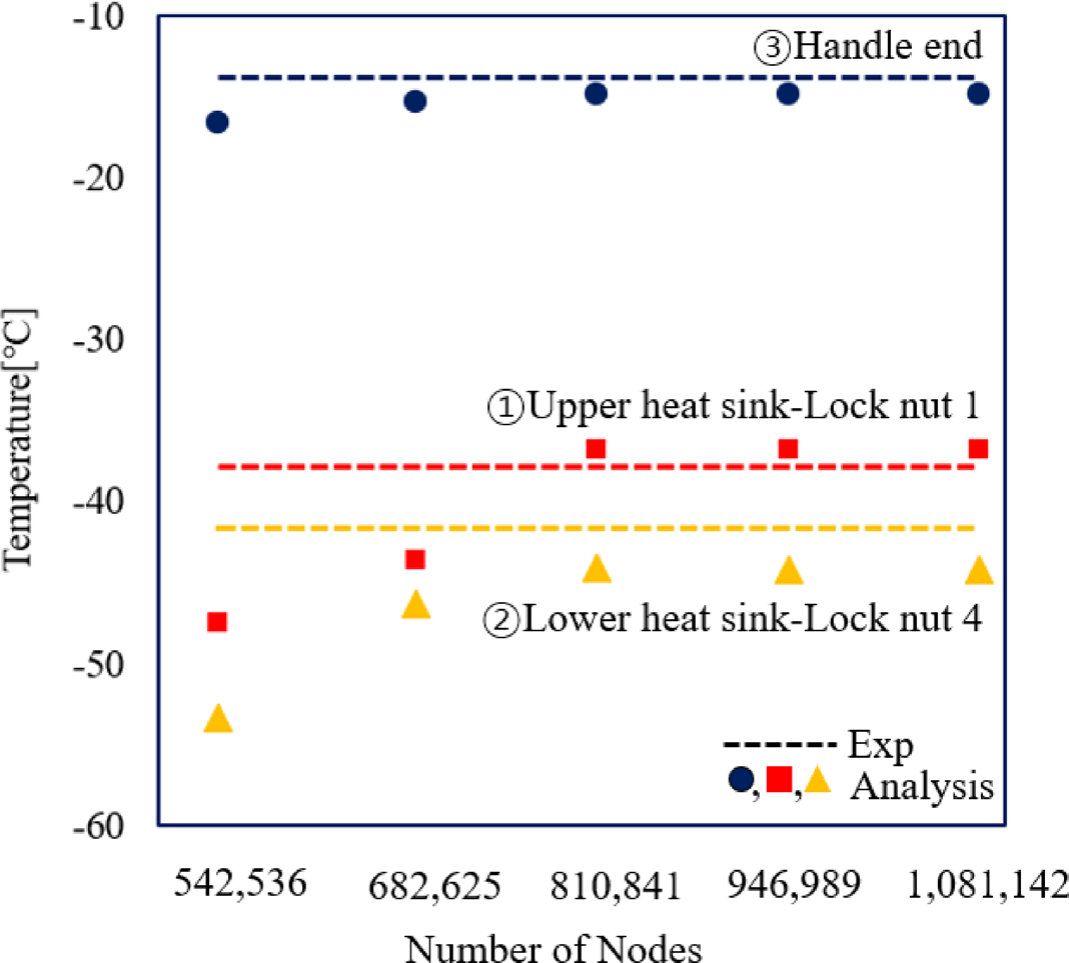
Mesh convergence test and comparison with experimental results in the heat transfer analysis.

A mesh convergence test was performed to ensure that the simulation results were independent of mesh density. Figure 5 shows the temperature variation at the handle end, upper heat sink and lower heat sink with respect to the number of nodes ranging from 542,536 to 1,081,142. The temperature values became nearly constant beyond approximately 810,841 nodes, showing low deviation. Therefore, the model with 810,841 nodes was adopted for subsequent analyses, as it provided stable temperature results and the closest agreement with the experimental data.

**Fig. 6:**
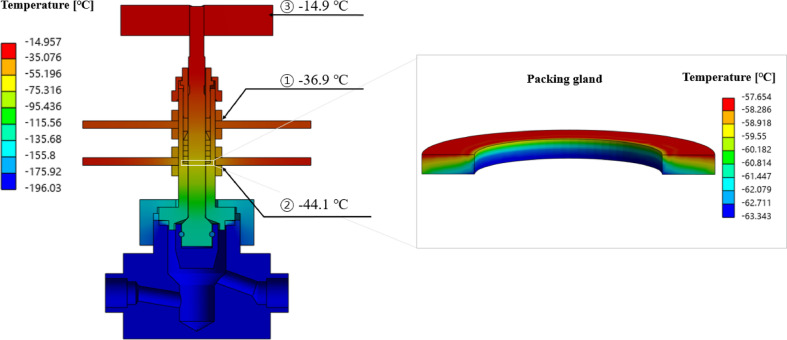
Results of heat transfer analysis.

Figure 6 shows the temperature distribution of the compact-type needle valve obtained from the heat transfer analysis under saturated conditions such as stabilized temperature during the cryogenic environment test. The temperature at the body and disc around the flow path, which were in direct contact with the liquid nitrogen, was found to be − 196 °C, and it was observed that areas farther from the flow path exhibited higher temperatures. Among the results obtained from the heat transfer analysis, the temperatures at the specific locations of temperature measurement for the cryogenic environment test were − 36.9 °C at the contact point between the upper heat sink and lock nut 1 (①), − 44.1 °C at the contact point between the lower heat sink and lock nut 4 (②), and − 14.9 °C at the end of the handle (③), showing differences of 2.6%, 5.7%, and 7.9% compared to the results of the cryogenic environment test, respectively. The error range was within 10%, indicating good agreement between the heat transfer analysis and cryogenic environment test. It was confirmed that the numerical analysis model constructed for evaluating the heat transfer characteristics estimates accurately the thermal behavior of the compact-type needle valve.

Meanwhile, the temperature distribution around the packing gland was estimated, which directly affects leakage, and presents as detailed results in Fig. 6 The temperature range around the packing gland was between − 63.3 °C and − 57.7 °C, with the lowest temperature of − 63.3 °C observed at the lower part. As shown in Table 1, the packing gland material is PEEK, which has the limit operating temperature of − 70 °C^15^, indicating that leakage prevention could be possible.

By introducing the pair of heat sinks installed on the upper and lower parts of the stem, leakage prevention was achieved, and the compact-type needle valve was designed with a bonnet length reduced to 85 mm compared to the conventional extended bonnet structure. However, although the outer diameter of the heat sinks, 150 mm, does not exceed the face-to-face distance standard of the compact-type needle valve, their relatively large size compared to the valve body may cause spatial constraints during installation. Therefore, size optimization is required to take into account of the outer diameter and thickness of the heat sinks as shape design variables while satisfying the requirement of the limit operating temperature for the packing gland.

## Size optimal design

### Parametric study

A parametric study on shape design variables was conducted to minimize the volume of the heat sink while satisfying the limit operating temperature of the packing gland material PEEK used for leakage prevention in the compact-type needle valve. The shape design variables were adopted as the outer diameter D and thickness t of the heat sink, while the inner diameter D_in_, which connects to the bonnet, was fixed at 24 mm. The combinations of the shape design variables for the parametric study were generated using the Latin Hypercube Sampling method^16^, resulting in a total of 25 combinations. In this study, D was limited to 150 mm or less to avoid exceeding the face-to-face distance of the compact-type needle valve, and set to 68 mm or more to ensure it exceeded the minimum width of the valve body. The thickness t was set within the range of 5 mm, which is the minimum manufacturable thickness, to 10 mm, which allows for the installation of the pair of the heat sinks. For each combination of design variables, heat transfer analysis was performed using the same loads and boundary conditions as described in Ch. 3.2, and the lowest temperature of the packing gland was evaluated. Table 3 summarizes the 25 combinations of shape design variables and the lowest temperatures of the packing gland obtained through the heat transfer analysis.

**Table 3:** Results of parametric study considering 25 combinations of shape design variables and lowest temperatures of packing gland obtained by heat transfer analysis.

Combination	D	t	Volume[mm^3^]	Lowest temperature of packing gland [°C]
1	75.58	5	20,160	–84.0
2	78.08	10	43,341	–78.0
3	105.81	6.34	52,867	–66.7
4	90.06	6.45	38,178	–74.1
5	85.53	10	52,914	–73.9
6	68.34	7.89	25,364	–87.9
7	107.70	6.79	58,809	–64.9
8	116.01	8.32	84,141	–60.3
9	95.81	8.01	54,114	–69.9
10	112.03	8.74	82,257	–58.2
11	117.64	7.32	76,283	–60.5
12	98.16	5.19	36,946	–71.4
13	141.94	9.56	146,995	–51.2
14	77.55	7.63	32,601	–72.9
15	92.28	8.92	55,650	–68.9
16	71.72	5.29	18,987	–87.3
17	134.62	5.57	76,773	–57.9
18	139.82	5.75	85,712	–56.4
19	123.83	9.72	112,703	–56.5
20	129.01	6.95	87,674	–57.4
21	131.30	7.46	97,632	–56.3
22	83.69	7.08	35,767	–77.4
23	113.08	5.93	56,903	–58.9
24	145.11	8.46	136,140	–64.9
25	149.25	9.82	167,419	–49.8

### Surrogate models

A surrogate model was constructed to estimate the lowest temperature of the packing gland at arbitrary values of the heat sink’s outer diameter D and thickness t, based on the lowest temperatures obtained from the 25 combinations derived from the parametric study. For this purpose, Kriging, a statistical estimation method, and ANN (Artificial Neural Network), which is based on training input-output data, were used.

Kriging is a method that estimates the value at an unobserved location as a linear weighted sum of nearby observed values^17^. Based on the DACE (Design and Analysis of Computer Experiments) method proposed by Sacks^18^, the surrogate model can be expressed as follows:

1 $$\:\widehat{y}\left(\mathrm{x}\right)=\mathrm{f}{\left(\mathrm{x}\right)}^{\mathrm{T}}\widehat{{\upbeta\:}}+{\mathrm{r}}^{\mathrm{T}}{\mathrm{R}}^{-1}(\mathrm{y}-\mathrm{F}\widehat{{\upbeta\:}})$$

$\:\widehat{y}\left(\mathrm{x}\right)$ is the predicted response at the target point x, f(x) is the regression function at that point, and $\:\widehat{{\upbeta\:}}$ is the regression coefficient estimated using generalized least squares. R represents the correlation matrix between observation points, while r is the correlation vector between the prediction point and each observation point. Matrix F consists of regression function values at all observation points, and y is the vector of observed response values. Equation (1) consists of two terms: the first term reflects the global trend estimated by the regression model, while the second term interpolates the residuals derived from the observations based on the correlation structure. In this study, the above surrogate model was implemented using Python, and RBF (Radial Basis Function) kernel^19^ was applied for the calculation of R and r. The surrogate model was obtained by training on the lowest temperatures of the packing gland for the 25 combinations and was used to estimate the lowest temperatures with respect to shape design variables D and t.

Meanwhile, ANN was structured with an input layer considering the shape design variables D and t, two hidden layers for training and validation, and an output layer that predicts the estimated lowest temperatures of the packing gland. Each hidden layer consists of three nodes, with non-linear activation functions applied to each node to enable effective training of the complex non-linear relationships between input and output variables. In the training process of the ANN, 70% of the total data obtained from the heat transfer analysis was used for training, while the remaining 30% was used for validation. The commercial software JMP Pro^20^ was used for both training and validation. The non-linear activation functions applied to each hidden layer and the output layer are summarized in Table 4.

**Table 4:** N0n-linear activation functions used in the two hidden and output layers of ANN.

Hidden Layer 1	H1	$\:\mathrm{T}\mathrm{a}\mathrm{n}\mathrm{h}(0.5\times\:(-0.015\times\:\mathrm{D}-0.229\times\:\mathrm{t}+3.442\left)\right)$
	H2	$\:\mathrm{T}\mathrm{a}\mathrm{n}\mathrm{h}(0.5\times\:(0.013\times\:\mathrm{D}-0.320\times\:\mathrm{t}-3.389\left)\right)$
	H3	$\:\mathrm{T}\mathrm{a}\mathrm{n}\mathrm{h}(0.5\times\:(0.039\times\:\mathrm{D}-1.247\times\:\mathrm{t}+5.748\left)\right)$
Hidden Layer 2	HH1	$\:\mathrm{T}\mathrm{a}\mathrm{n}\mathrm{h}(0.5\times\:(-0.530\times\:\mathrm{H}1-0.759\times\:\mathrm{H}2-3.24\times\:\mathrm{H}3-0.079\left)\right)$
	HH2	$\:\mathrm{T}\mathrm{a}\mathrm{n}\mathrm{h}(0.5\times\:(1.218\times\:\mathrm{H}1-1.191\times\:\mathrm{H}2-1.197\times\:\mathrm{H}3-0.629\left)\right)$
	HH3	$\:\mathrm{T}\mathrm{a}\mathrm{n}\mathrm{h}(0.5\times\:(-3.589\times\:\mathrm{H}1-0.008\times\:\mathrm{H}2-0.691\times\:\mathrm{H}3-0.479)$
Output Layer	Output	$\:32.7173\times\:\mathrm{H}\mathrm{H}1-84.481\times\:\mathrm{H}\mathrm{H}2-26.164\times\:\mathrm{H}\mathrm{H}3-102.775$

**Fig. 7:**
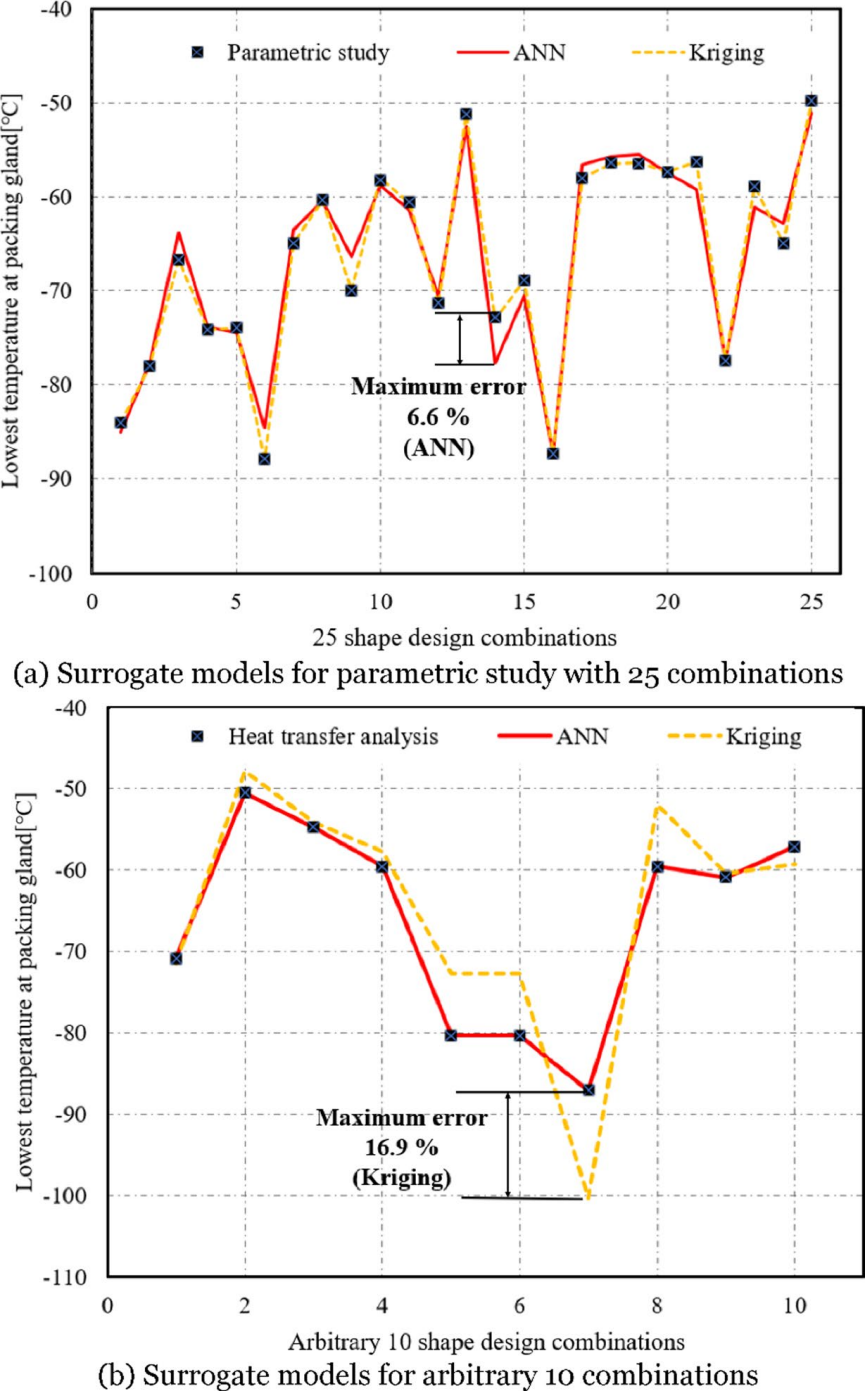
Lowest temperatures at packing gland estimated by surrogate models using ANN and Kriging in comparison with that from parametric study with 25 combinations of shape design variables and heat transfer analysis at arbitrary 10 combinations.

The surrogate models that estimated the lowest temperature of the packing gland for the 25 combinations of the shape design variables D and t using Kriging and ANN methods, are shown in Fig. 7a. Both surrogate models accurately estimated the lowest temperature of the packing gland obtained from the parametric study. Regarding the maximum error between the two methods, Kriging showed a maximum error of 0.28%, and ANN showed 6.6%, indicating a high correlation with maximum errors below 10%. Meanwhile, Fig. 7b shows the results of applying the surrogate models to 10 arbitrary combinations not included in the 25 combinations used in the parametric study. The ANN method maintained a maximum error of 9.4%, demonstrating prediction accuracy within 10%, while the Kriging method showed a maximum error of 16.9%, indicating reduced prediction accuracy at shape design variables outside the 25 combinations. These are attributed to the training mechanisms. The ANN method utilized a portion of the training data for validation, helping to generalize predictions, whereas the Kriging method estimated correlations solely based on the observed shape design variables, leading to reduced prediction accuracy at arbitrary shape design variables beyond that of 25 combinations^21,22^.

### Design space exploration

Using the surrogate model obtained through the ANN method, the design space of the shape design variables was explored to minimize the volume of the heat sink while satisfying the limit operating temperature of the packing gland material PEEK, which is essential for leakage prevention in the compact-type needle valve. The design space exploration was conducted based on the following size optimization formula:

Minimize volume of heat sink:

$$\:\mathrm{V}=\frac{{\uppi\:}}{4}({\mathrm{D}}^{2}-{\mathrm{D}}_{\mathrm{i}\mathrm{n}}^{2})\times\:\mathrm{t}$$

Subject to: $$\:68\:\mathrm{m}\mathrm{m}\le\:\mathrm{D}\le\:150\:\mathrm{m}\mathrm{m}$$

$$\:5\:\mathrm{m}\mathrm{m}\le\:\mathrm{t}\le\:10\:\mathrm{m}\mathrm{m}$$

$$\:{\mathrm{T}}_{\mathrm{P}\mathrm{a}\mathrm{c}\mathrm{k}\mathrm{i}\mathrm{n}\mathrm{g}\:\mathrm{g}\mathrm{l}\mathrm{a}\mathrm{n}\mathrm{d}}\ge\:{\mathrm{T}}_{\mathrm{L}\mathrm{i}\mathrm{m}\mathrm{i}\mathrm{t}\:\mathrm{o}\mathrm{f}\:\mathrm{P}\mathrm{E}\mathrm{E}\mathrm{K}}(=-70\:^\circ\:\mathrm{C})$$

The objective function was defined as the minimization of the heat sink volume. The ranges of the shape design variables D and t were set the same as in the parametric study, and a constraint was applied to ensure that the lowest temperature of the packing gland exceeds the limit operating temperature of PEEK, which is − 70 °C.

**Fig. 8:**
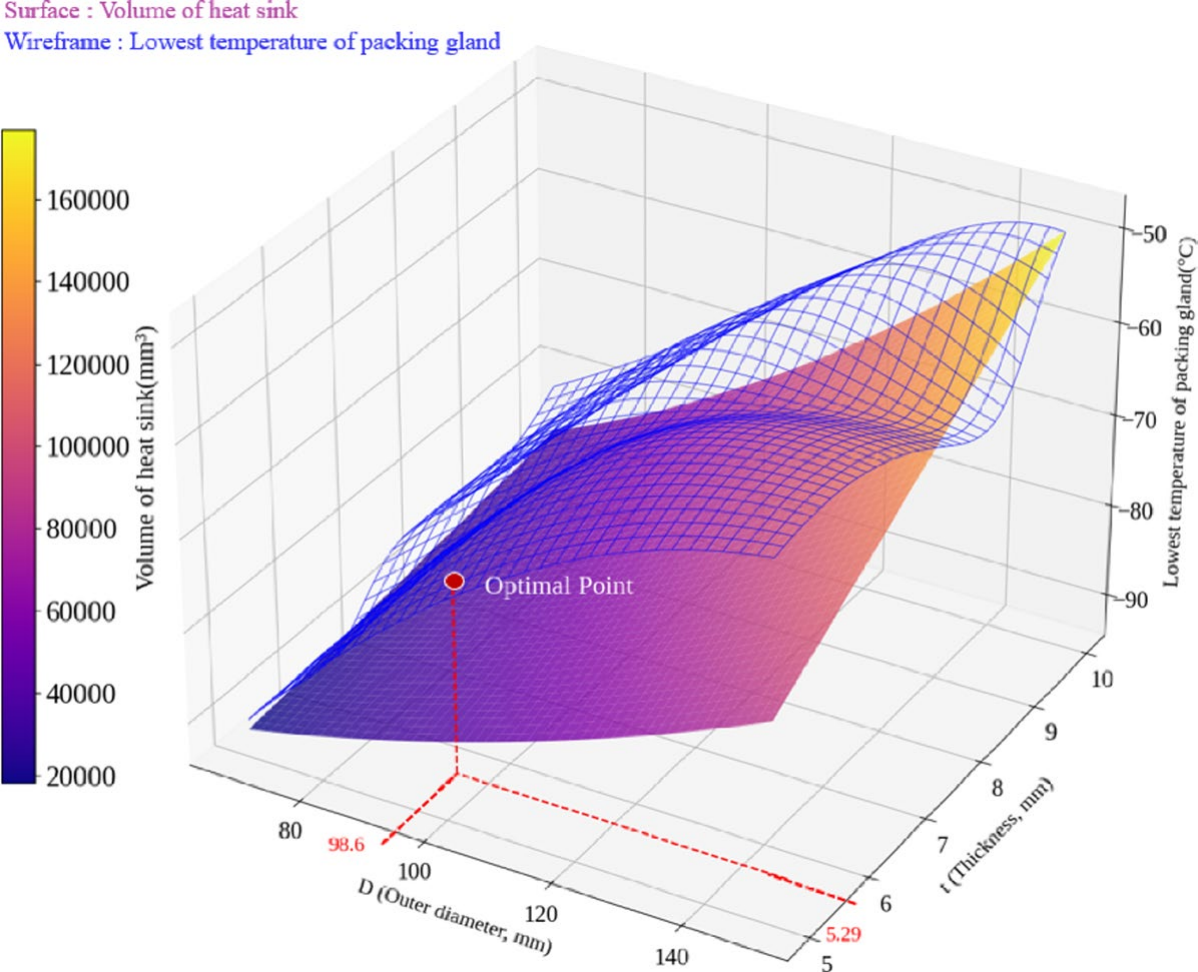
Results of design space exploration to find D and t regarding to volume of heat sink and lowest temperature of packing gland.

Figure 8 shows the results of the design space exploration, illustrating the correlation between the volume of heat sink and the lowest temperature of packing gland regarding to changes in D and t. The volume of heat sink is visualized as a surface, while the lowest temperature of packing gland is represented as a wireframe. As D and t increase, the volume of heat sink increases across the entire design space, and the lower temperature of packing gland increases, reducing the risk of freezing. This presents a trade-off, in which the volume of heat sink increases, as the risk of freezing decreases. Therefore, an optimal point was searched for where the volume of heat sink is minimized while satisfying the limit operating temperature of the packing gland material. The optimal values of D and t were found to be 98.6 mm and 5.29 mm, respectively, with the lowest temperature of the packing gland at –69.9 °C, and the corresponding volume was 37,899 mm^3^. The optimized outer diameter D of the heat sink is more than 30% smaller than the initial model’s 150 mm, which helps avoid spatial constraints for the installation of the compact-type needle valve, and the volume of heat sink was also reduced by over 54%.

**Fig. 9:**
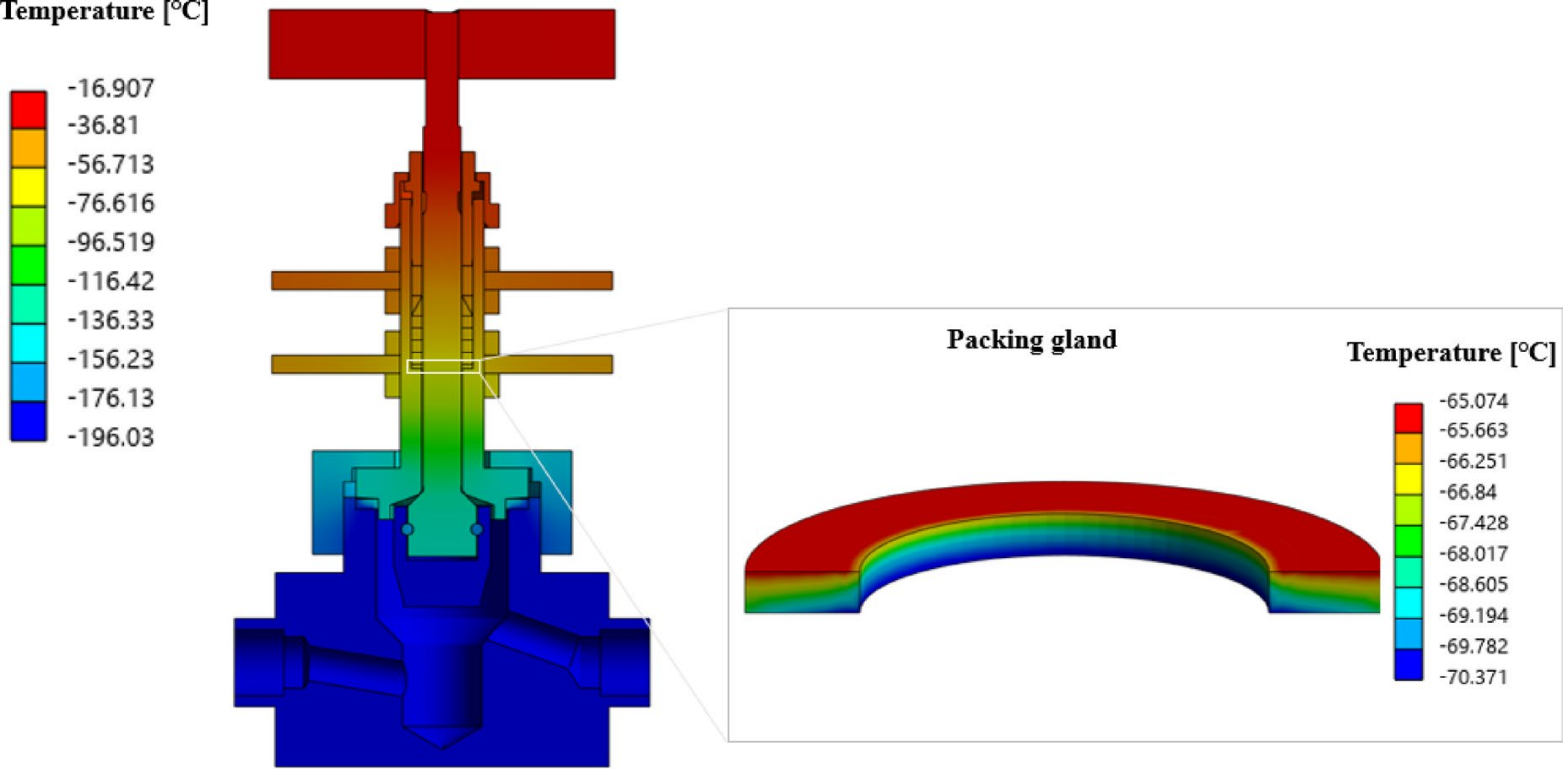
Results of heat transfer analysis for optimized model.

Figure 9 shows the results of the heat transfer analysis conducted on the compact-type needle valve with heat sinks applied by the optimized D and t obtained from the design space exploration, to verify the results of the size optimization. The lowest temperature of the packing gland was calculated as − 70.3 °C, which shows a 0.56% error compared to the.

–69.9 °C predicted by the size optimization, confirming good agreement between them.

## Cryogenic leakage test

A prototype of the compact-type needle valve was manufactured using the optimized shape design variables D and t obtained through the design space exploration. Considering manufacturable dimensions in the fabrication process, D and t were set to 98 mm and 6 mm, respectively. To determine leakage, a cryogenic leakage test according to BS 6364^12^ was conducted on the manufactured prototype. The cryogenic leakage test consists of four parts: pressure test, leak-tightness test, low-pressure endurance test, and ambient temperature recovery test. Among these, the leak-tightness test was performed.

**Fig. 10:**
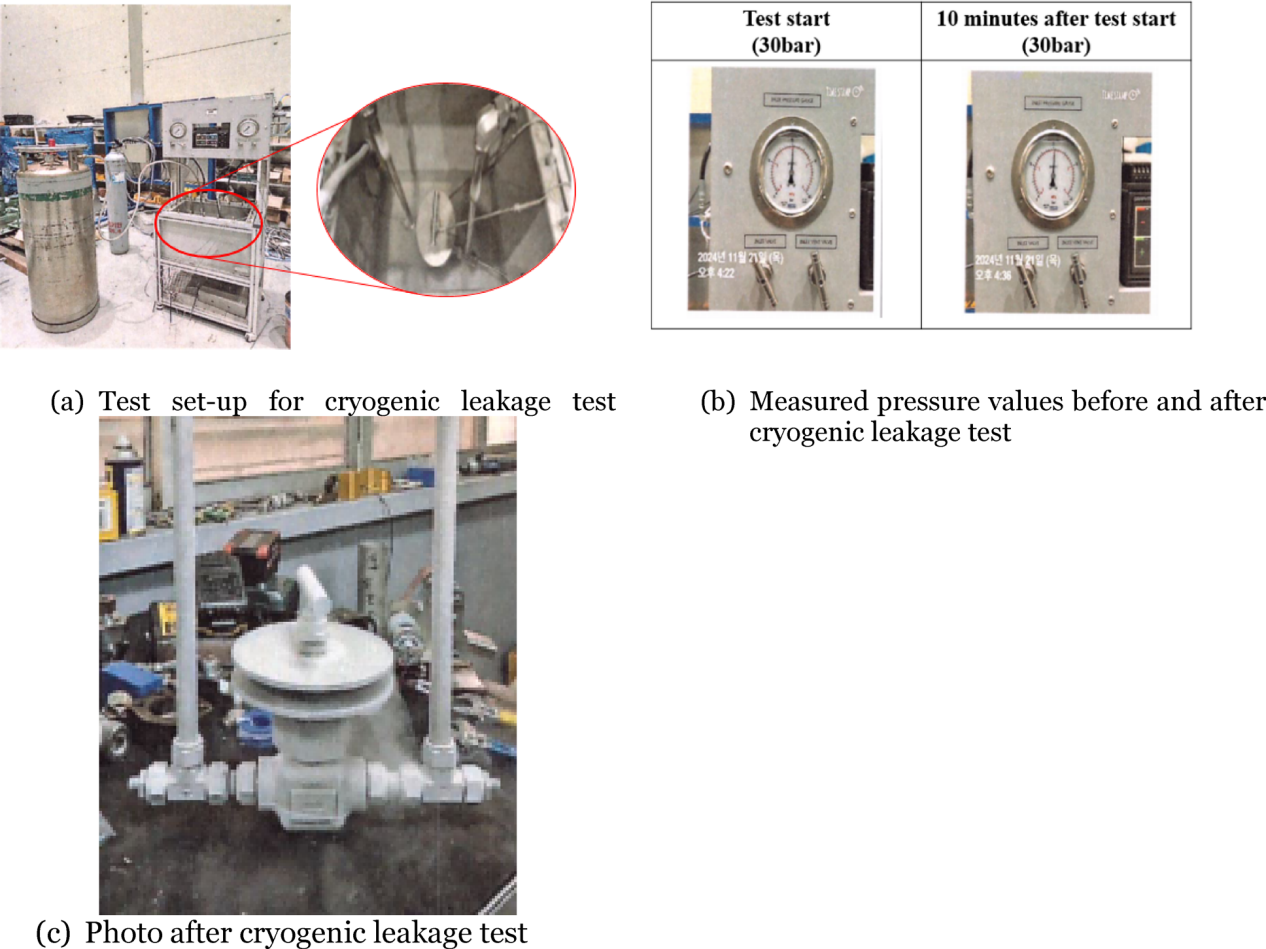
Cryogenic leakage test for compact-type needle valve with optimized shape design variables.

Figure 10 shows the configuration of the test equipment installed for the cryogenic leakage test and the test procedure itself. As shown in Fig. 10a, the prototype of the compact-type needle valve was placed inside a cryogenic chamber, filled with liquid nitrogen up to the point where the union nut located at the top of the valve body was submerged, and maintained for more than one hour until the temperature inside the prototype stabilized. Afterward, with the outlet end of the prototype valve closed, helium gas was injected at 20 bar and 30 bar—equivalent to 110% and 150% of the pressure rate—into the inlet and maintained for 10 min, followed by 100 cycles of pressurization. Leakage of helium gas was checked based on pressure readings measured by the pressure gauge installed at the outlet. Figure 10b shows the pressure readings measured at the outlet: the readings were taken after injecting 30 bar of helium gas and maintaining it for 10 min, as well as after completing 100 cycles of pressurization. No change was observed between the two measured values, confirming compliance with the requirements of BS 6364. Figure 10c shows the appearance of the prototype of compact-type needle valve after the cryogenic leakage test, confirming that no leakage occurred in the valve with the heat sinks applied by the optimized shape design variables.

## Conclusions

In this study, a cryogenic environment test, along with heat transfer analysis, was conducted on a compact-type cryogenic needle valve to evaluate the thermal behavior of its components. A parametric study and size optimization were performed by treating the heat sink’s outer diameter and thickness as shape design variables, with the objective of minimizing the heat sink’s volume. The results obtained are as follows:

1. In the cryogenic environment test using liquid nitrogen as the working fluid, temperatures were measured at three locations after one hour from the start of the test: − 41.7 °C at the contact point between the lower heat sink and lock nut 4,
–37.9 °C at the contact point between the upper heat sink and lock nut 1, and.
–13.8 °C at the handle end. The numerical results obtained from the heat transfer analysis, conducted under the same loads and boundary conditions as the test, showed agreement with the test results within a maximum error of 7.9%.
4. From the heat transfer analysis, the lowest temperature of the packing gland—which directly affects leakage—was estimated to be − 63.3 °C. Since this value is above the limit operating temperature of the packing material (–70 °C), leakage is expected to be effectively prevented.
5. To mitigate spatial constraints caused by the relatively large heat sink compared to the valve body, a parametric study was performed using the outer diameter D and thickness t of the heat sink as shape design variables. The study included 25 combinations of the shape design variables, each considering the resulting lowest temperature of the packing gland. Based on the results, an artificial neural network-based surrogate model was constructed to predict the packing gland’s lowest temperature. Using this surrogate model, design space exploration was conducted to determine the optimal values of D and t for minimizing the volume of heat sink.
6. As a result of the design space exploration, the optimal values of D and t were found to be 98.6 mm and 5.3 mm, respectively. Consequently, the heat sink volume was reduced to 54% of the initial model. A cryogenic leakage test, conducted on the prototype equipped with the optimized heat sink in accordance with BS 6364, confirmed that no leakage occurred, thereby verifying compliance with the standard.

## Data Availability

The datasets analyzed during the current study are available from the corresponding author upon reasonable request.
